# Biodeterioration of Microplastics by Bacteria Isolated from Mangrove Sediment

**DOI:** 10.3390/toxics11050432

**Published:** 2023-05-05

**Authors:** Shu-Yan Ren, Hong-Gang Ni

**Affiliations:** School of Urban Planning and Design, Peking University Shenzhen Graduate School, Shenzhen 518055, China; 2206394203@pku.edu.cn

**Keywords:** MPs, domestication, mechanism of biodegradation, 16S rDNA, mangrove sediment

## Abstract

As a kind of ubiquitous emerging pollutant, microplastics (MPs) are persistent in the environment and have a large impact on the ecosystem. Fortunately, some microorganisms in the natural environment can degrade these persistent MPs without creating secondary pollution. In this study, 11 different MPs were selected as carbon sources to screen the microorganisms for degradable MPs and explore the possible mechanism of degradation. After repeated domestication, a relatively stable microbial community was obtained after approximately 30 days later. At this time, the biomass of the medium ranged from 88 to 699 mg/L. The growth of bacteria with different MPs ranged from 0.030 to 0.090 optical density (OD) 600 of the first generation to 0.009–0.081 OD 600 of the third generation. The weight loss method was used to determine the biodegradation ratios of different MPs. The mass losses of polyhydroxybutyrate (PHB), polyethylene (PE), and polyhydroxyalkanoate (PHA) were relatively large, at 13.4%, 13.0%, and 12.7%, respectively; these figures for polyvinyl chloride (PVC) and polystyrene (PS) were relatively slight, 8.90% and 9.10%, respectively. The degradation half-life (t_1/2_) of 11 kinds of MPs ranges from 67 to 116 days. Among the mixed strains, *Pseudomonas* sp., *Pandoraea* sp., and *Dyella* sp. grew well. The possible degradation mechanism is that such microbial aggregates can adhere to the surface of MPs and form complex biofilms, secrete extracellular and intracellular enzymes, etc., break the hydrolyzable chemical bonds or ends of molecular chains by attacking the plastic molecular chains, and produce monomers, dimers, and other oligomers, leading to the reduction of the molecular weight of the plastic itself.

## 1. Introduction

As one of the most common synthetic materials [1], global output of plastics has soared from 150 tons in 1950 to 391 million tons in 2021 [2]. Consequently, a huge volume of plastic waste infiltrates the environment each year. However, only 28% of plastics are recycled or incinerated [3]. Plastics can break into small pieces in the environment, ultimately forming stable small particles with a diameter of less than 5 mm that are known as microplastics (MPs) [4,5]. Generally, MPs include plastic fragments, particles, and textile fibers [6].

MPs are ubiquitous and are detected in the atmosphere, water, soil, and other environmental media [7,8,9,10]. The accumulation of MPs in the environment has adverse effects on organisms, soil, and water. In particular, widespread MP infiltration is a global concern for the aquatic environment, posing a threat to existing life forms. For example, MPs in aquatic ecosystems can lead to false satiety, pathological stress, reduced growth rates, and reproductive complications [11,12,13]. In addition, MPs easily bind to other toxic chemicals or metals, acting as vectors for such toxic substances [14] and causing synergetic pollution effects [15,16]. To control the ecological risk of MPs, the decontamination of MPs in the environment is crucial.

Microbial degradation is a promising ecofriendly method for the removal of MPs with no harm to the environment [17]. Therefore, screening and identifying microorganisms that can degrade different types of MPs is conducive to the natural bioremediation process and the cleaning of the natural ecosystems. Most microorganisms are opportunistic and have inherent adaptability. They can adapt to every environment they find and have the potential to transform a variety of compounds, including plastic polymers [18]. During polymer degradation, microorganisms first adhere to the polymer surface, subjecting it to microbial colonization. Then, the microorganisms will secrete extracellular enzymes that bind to the polymer and cause hydrolysis and cleavage [19]. After that, the polymer is degraded into a low-weight polymer and mineralized into carbon dioxide and water for microorganisms to use as energy [20]. It has been found that certain microorganisms can degrade certain plastic particles. For example, polypropylene (PP) can be degraded by *Rhizopus arrhizus* [21] and *Vibrio* and *Pseudomonas* sp. [22]; *Bacillus* sp. can degrade polyethylene (PE) [18] and *Klebsiella*, *Citrobacter*, and other bacteria can degrade polystyrene (PS) [23].

The high temperature, high salinity, pH value, organic matter level, and low ventilation and humidity of mangrove sediment improve the substrate conditions to be conducive to the growth of a variety of microorganisms [24,25]. Additionally, mangroves can also improve the availability of a wide range of microorganisms with multiple types of potential. To the best of our knowledge, microbial degradation of different kinds of MPs has rarely been observed simultaneously. On that basis, this study was aimed at demonstrating the growth and biodegradation ability of bacterial isolates from mangrove sediment in the degradation of 11 kinds of MPs. The degradation degree of MPs in the sample can be examined according to the change in mass and the change in optical density (OD) value. 16S rDNA technology was used to identify microorganisms that can degrade different kinds of MPs. This study attempts to identify a remedial option for the accumulation of MPs in the marine environment and has certain significance for ecological restoration.

## 2. Materials and Methods

### 2.1. Chemicals

A total of 11 kinds of MPs—polyamide (PA), poly(butylene succinate) (PBS), polycaprolactone (PCL), polyethylene (PE), polyhydroxyalkanoates (PHA), polyhydroxybutyrate (PHB), polylactic acid (PLA), polypropylene (PP), polystyrene (PS), polyvinyl alcohol (PVA), and polyvinyl chloride (PVC)—were selected as carbon sources to screen microorganisms (Zhejiang Shangyu Yixin Ball Industry, Shaoxing, China). The particle size of the abovementioned MPs was determined by a 1000 mesh (13 μm). Additionally, disodium phosphate (Na_2_HPO_4_), sodium dihydrogen phosphate (NaH_2_PO_4_) and ammonium dihydrogen phosphate (NH_4_H_2_PO_4_) were added to cultivate microorganisms.

### 2.2. Sample Collection and Screening of MP-Degrading Bacteria

Sediment samples were collected in the mangrove area of Shenzhen, China (114°03′ E, 22°32′ N). A sterile shovel was used to collect samples from a sediment depth of 1 to 5 cm. Different MP beads, NH_4_H_2_PO_4_, and O_2_ were used as the sole sources of carbon, nitrogen, and oxygen for culturing microorganisms. A liquid mixture of NH_4_H_2_PO_4_ (5.00 g/L), sediment (67.0 g/L), and different MPs (1.00 g/L) was used to cultivate and domesticate microorganisms in mangrove sediment (pH 6.50–7.50, regulated by Na_2_HPO_4_ and NaH_2_PO_4_). Additionally, a blank cross reference was set. For the first generation of culture, 50.0 mL of liquid mixture was domesticated in the incubator shaker at ~29 ℃ at a vibration speed of 150 rpm [18].

Then, the cells were transferred to another fresh MPs−inorganic salt medium. An inoculation of 10% was conducted every 10 days. The weight of the medium and its OD 600 (0.05 < OD 600 < 0.90) were measured. For the blank control, the uninoculated medium without MPs was kept under similar conditions. This process was repeated until the microbial culture was adapted to the specific environment. After subculture was conducted three times, the relative abundance of operational taxonomic units (OTUs) of some strains in the growth medium increased significantly, while the concentration of MPs decreased. The main degrading microorganisms come from the third generation of bacterial suspensions.

### 2.3. Microbial Degradation of MPs

The mass change of the culture medium can reflect the degradation of the MPs to a certain extent. A four decimal balance (AL204-IC, Mettler Toledo, Shanghai, China) was used to weigh the mass change [26]. The turbidity method uses the OD (0.05 < OD 600 < 0.90) value to characterize the growth curve of microorganisms [27]. The OD 600 value mirrors the growth trend of microorganisms measured by ultraviolet spectrophotometry. The biomass (mg/L) was recorded as b, and the absorption coefficient was recorded as x. Then, the biomass of the microorganisms in the bacterial suspension was estimated by Equation (1) according to the OD value [28]:

ln b = −1.05 ln x + 4.26(1)

The weight loss of the MPs as a percentage was determined using Equation (2):

(2) $$weight\;loss\;(\%)=\frac{\left(W_{0}-W\right)}{W_{0}}$$

where the initial weight and current weight were designated as W_0_ and W, respectively. The data were further processed to determine the rate constant of MP reduction by using a first-order kinetic model (Equation (3)) based on initial and final weights at a specific time interval (10 days) [29]:

(3) $$k=-\frac{1}{t}\left(ln\frac{W}{W_{0}}\right)$$

### 2.4. 16S rDNA

The total genomic DNA in the samples was extracted using a Soil DNA Kit. DNA (20.0–30.0 ng) was used to produce amplicons and the concentration was monitored with a Qubit 3.0 Fluorometer. The V3 and V4 hypervariable regions of prokaryotic 16S rDNA were selected to generate amplicons and perform classification analysis. Additionally, the index adapter was added to the end of the 16S rDNA amplicon to generate index libraries and prepare for the upstream and downstream next-generation Illumina sequencing. During the sequencing process, Illumina’s built-in software was used to determine whether to retain or discard each sequencing fragment, i.e., read, based on the quantity of the first 25 bases. The original sequencing data (pass filter data) were obtained, and the results were stored in FASTQ file format, which includes sequencing information and the corresponding sequencing quantity information. Then, 1.00 μL of each primer, 2.00 μL dNTPs, 2.50 μL TransStart Buffer, and 20.0 ng template DNA were used to carry out a chain reaction in a mixture of 25.0 μL. These polymerase chain reactions were performed in triplicate.

Double-end sequencing was conducted and the positive and negative reads of the first of the two sequences were connected together to carry out filtering, with the results contained in the N sequence being joined together to retain the sequence lengths larger than 200 bp. The chimeric sequence was filtered and purified, and the obtained OTU cluster sequence was subjected to clustering with VSEARCH clustered (1.9.6) (sequence similarity was set to 97%). Moreover, the 16S rDNA reference database used was Silva 132. Additionally, the representative sequences were analyzed using the ribosomal database program classifier Bayesian algorithm for OTU species classification, and the community composition of each sample was determined on the basis of the statistics of different species classification levels.

## 3. Results and Discussion

### 3.1. Bacterial Screening and Identification

Compared with other degradable materials, plastics are not susceptible to microbial attack [30]. However, MPs provide a new niche for microorganisms, support the colonization and growth of microorganisms, and serve as a carbon source. To explore the degradation behavior of MPs, different kinds of MP-degrading microorganisms were screened from mangrove sediment.

**Figure 1:**
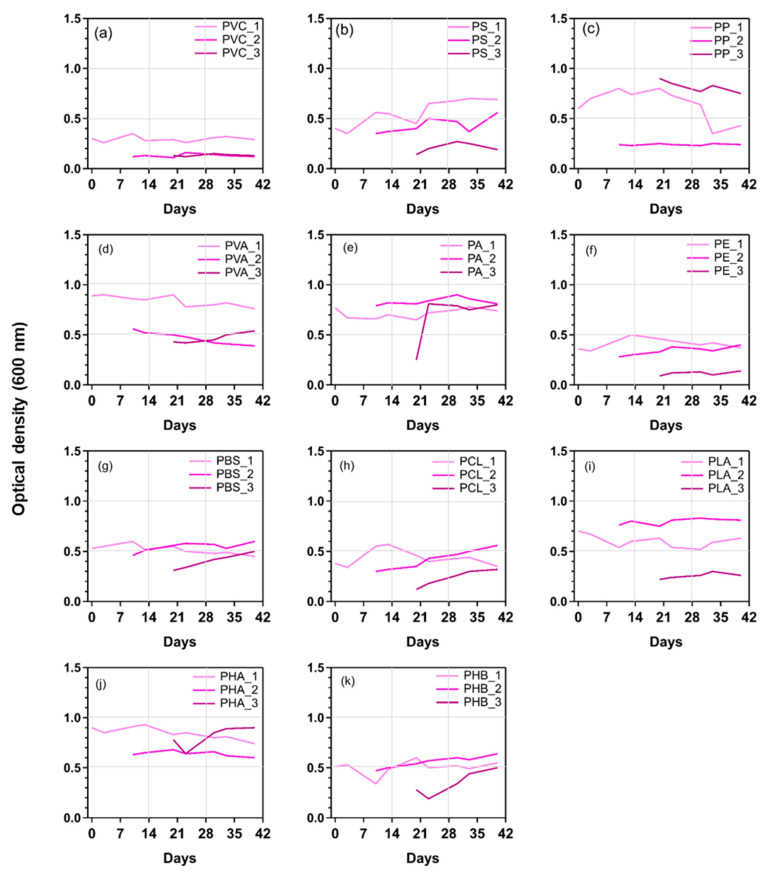
Change in OD values of culture liquid with various MPs as carbon sources. The line colors from light to dark represent the first generation to the third generation of domestication. Subfigures (**a**–**k**) represents the changes in 11 types of MPs.

Optical density, reflecting the growth of strains, is widely used to estimate the concentration of cells in liquid cultures [18]. Figure 1 shows the growth curve of microorganisms cultured with different kinds of MPs as the sole carbon source.

In the first three days, the growth of microorganisms usually has a lag time for the microbial OD value, during which the OD value basically decreases rather than increases. The reason for this phenomenon is that cell proliferation is required to synthesize various substances and undergo a process of adjustment and adaptation [28]. Within 4–5 days of culture, the microorganisms showed signs of growth around the polymer particles and began to attach to the polymer particles. After 10 days, the OD of the bacterial suspension fluctuated, and the microorganisms extended around the particles within 10–15 days, when the microbial growth rate was the highest. At this stage (the growth regulation period), the enzyme system was active, and metabolism was vigorous. No growth was observed on the control sample. After repeated domestication, the ability to degrade MPs was stronger, the degrading strains were continuously enriched, and a relatively stable microbial community was formed approximately 30 days later. At this time, the biomass range was 88 mg/L (PP)–699 mg/L (PE). Specifically, the growth of bacteria with different MPs ranged from 0.30 to 0.90 in the first generation to 0.09–0.81 in the third generation indicating that mixed strains of microorganisms adapted to the culture conditions and used MPs as the corresponding carbon source for growth.

In general, the time trends for the OD values of different MPs were the same (Figure 1). Taking PP as an example, obvious growth was observed from Day 0 (0.60) to Day 10 (0.80) (Figure 1c). Our results were comparable to those of other studies [27]. The effect of a single strain on the degradation of PP was studied, and it was found that its OD value ranged from Day 0 (0.251) to Day 10 (0.903) [27]. However, the increase in the growth rate on Day 10 does not indicate a strong reaction and performance of microorganisms when exposed to MPs. The increase shows that this period was conducive to the interaction between the bacterial cell membrane and MPs, thus allowing rapid metabolism. From Day 20 to Day 30 after exposure to PP, it was observed that the growth of microorganisms decreased from 0.80 to 0.64. The decline in the OD value may have been caused by dilution of the culture medium, cell lysis, nutrient depletion, etc. [31]. Different types of MPs can be used as the sole carbon source to screen microorganisms with strong degradation capacity. It was not surprising that the concentration of microorganisms in the mixed bacterial suspension with different MPs as the sole carbon source was different. For example, the original structure of the microbial populations in mangrove sediment may be responsible for this.

### 3.2. Reduction Rate of MPs

**Figure 2:**
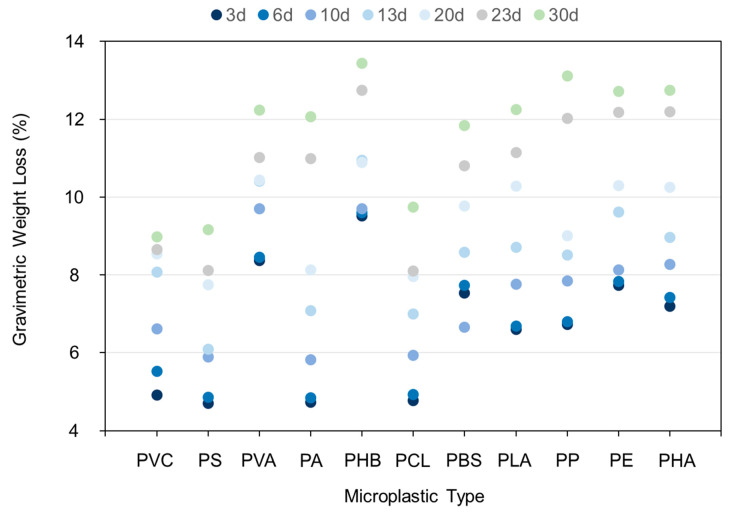
The mass reduction ratios of 11 kinds of MPs over time. The blue and green dots refer to the weight change after 3 days and 30 days of culture.

The weight loss of MPs after inoculation of mangrove mixed strains caused by microbial action was examined (Figure 2). With the increasing of culture time, the quantity of MPs decreased. However, there were obvious differences between different MPs. After 30 days of degradation, the gravimetric weight losses of PHB, PE, PHA, PVC, and PS were 13.4%, 13.0%, 12.7%, 8.90%, and 9.10%, respectively.

**Figure 3:**
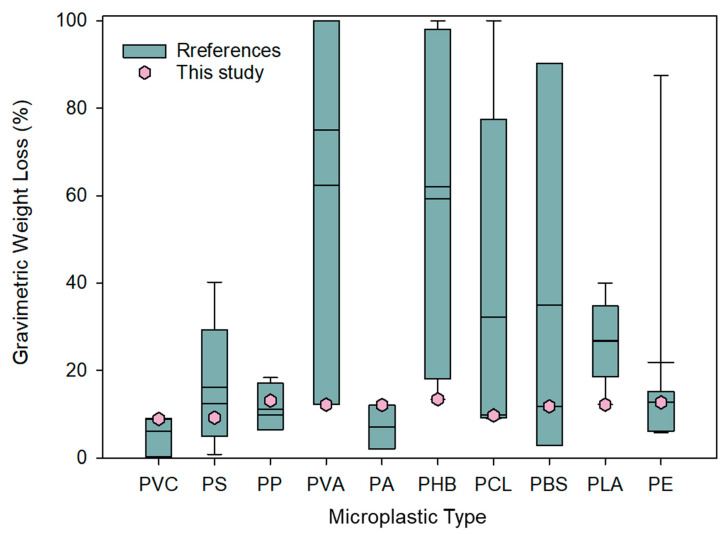
Comparison of this study with other studies. The boxes and dots display the degradation ratios of microplastics in the previous studies and in this study.

Our results (Table S1) were comparable with those of previous studies that obtained the gravimetric weight loss of PS (12.4%) and PE (7.50%) after 42 days and 56 days of degradation, respectively [32] (Figure 3). According to the present study, after 30 days of culture, the gravimetric weight loss of PS and PE reached 9.10% and 13.0%, respectively. These results were lower than that of PS degraded for 140 days (52.4%). A possible explanation may be that four strains that were most suitable for PE degradation were used in their study, and their degradation time was also much longer than ours [33]. In a previous study [27], two single strains were used to degrade PP, and the weight loss of PP was 6.40% and 4.00% after 40 days of culture. Our result (13.1%) was higher than these figures. This underscored that a mixed culture performs better than a single microbial culture in the environment [34]. PLA is a biodegradable plastic and its gravimetric weight loss reached 12.2%. A previous study [35] found that after incubation with *Stenotrophomonas maltophilia LB 2-3*, the molecular weight and tensile properties of PLA decreased rapidly.

**Figure 4:**
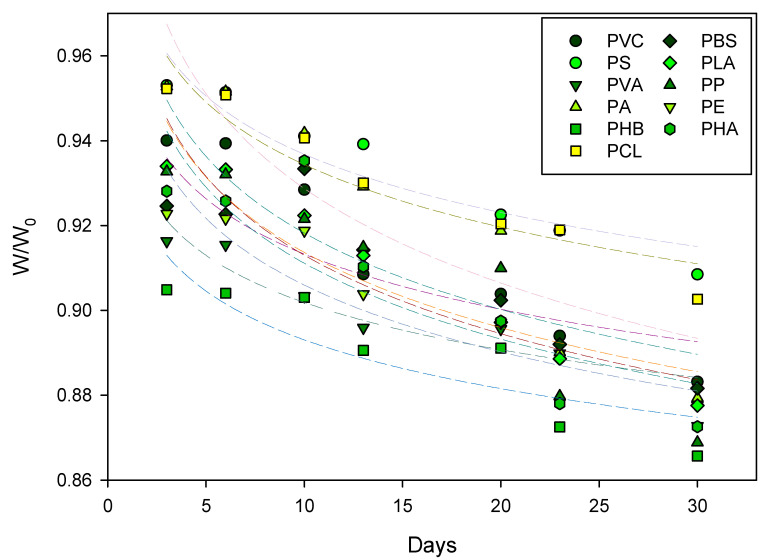
Degradation kinetics of 11 MPs in response to bacteria isolated from mangrove sediment. The different colored lines represent the degradation kinetics curves of 11 types of MPs.

Via dynamic simulation based on the experimental data, the biodegradation kinetic curves (Figure 4), kinetic equations, and corresponding coefficients of different MPs and the biodegradation half-life (t_1/2_) of different MPs were obtained (Table 1). In general, the fitted linear graph shows that the degradation data conform to the first-order kinetic equation, with an R^2^ of 0.70–0.90. According to mathematical statistical analysis, when the correlation coefficient R^2^ of the model is between 0.5 and 1, it is considered to indicate a strong correlation [36]. The K value of the 11 MPs was 0.006–0.010 (Table 1). Regarding the relative order of biodegradation difficulty, PA was the most difficult to degrade, followed by PS, and PHB was the easiest to degrade. For all 11 kinds of MPs involved in this study, the main difference lies in their carbon chain structure. For example, the main chain of polyolefin plastics (e.g., PS) is composed of a carbon skeleton with stable chemical properties and a low degradation rate [37]. However, polyester (e.g., PLA) contains ester bonds that are easy to hydrolyze and are easily biodegradable [38]. For example, the t_1/2_ of PLA was 85.76 days and that of PS was 114.1 days.

### 3.3. MP-Degrading Bacteria

**Figure 5:**
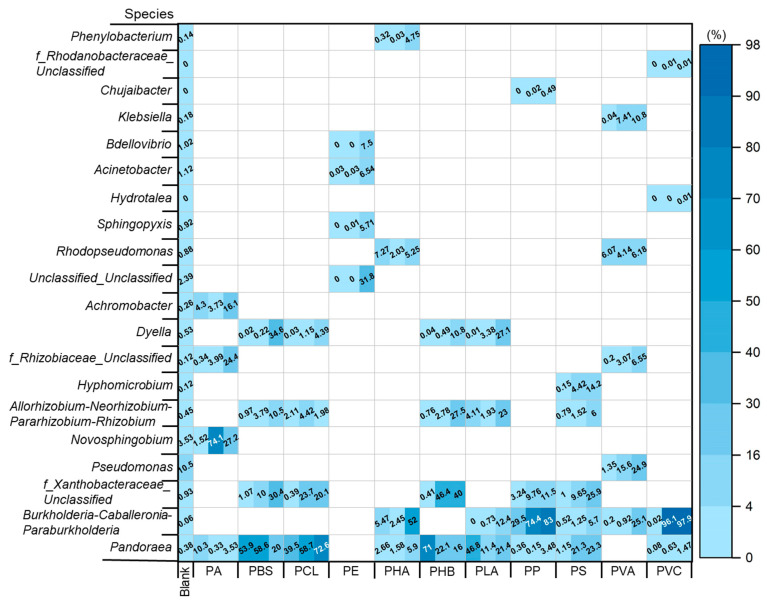
MP-relative abundance of degrading bacteria. The first column in the grid is the blank control and the next three columns correspond to the third generation of microorganisms.

16S rDNA detection of the microbial culture medium after 30 days of degradation provided species information on the MP-degrading bacteria (Figure 5). The results indicated that *Pseudomonas* sp., *Pandoraea* sp., and *Dyella* sp. could grow well in the mixed strains with the progress of domestication. These bacteria can make good use of a variety of organic and inorganic compounds, which enables them to live flexibly on the surface of MPs [39,40,41].

Overall, different MP-degrading bacteria isolated from different environmental media are usually different with different degradation abilities. Specifically, the PS mass decreased by 9.20% within 30 days under the effect of *Hyphomicrobium* sp. and *Pandoraea* sp. Other studies showed that PS weight could be reduced by 12.4–40.2% under the action of *Pseudozyma japonica Y7-09* [26,42]. Apparently, different bacteria that play a main role are responsible for this.

The main screened MP-degrading bacteria for PHB, PCL, and PVA in this study were *Allorhizobium-Neorhizobium-Pararhizobium-Rhizobium* sp., *Pandoraea* sp., and *Pseudomonas* sp. Under their influence, the weight of the two kinds of MPs decreased by 13.4% and 9.70% within 30 days, respectively. Previous studies found that PHB (*Pseudomonas mendocina DS04-T*) [43], PCL (*Micorbispora rosea* subsp. *Taiwanensis HS 45-1*) [44], and PVA (*Penicillium* sp. *WAH02-21*) [45] can reach 100% degradation in approximately 10 days under the action of some specific bacteria. Obviously, the degradation effect of mangrove sediment mixed flora on PHB is relatively poor. This could be attributed to different sources of bacteria. Our flora came from mangrove sediment, while *Micorbispora rosea* subsp. *Taiwanensis HS 45-1* and other bacteria were isolated from soil.

Previous studies found that PBS (14 days) and PE (60 days) can degrade more than 87% under the action of fungi (*Bacillus pumilus 1-A*) [46] and soil bacteria (*Bacillus cereus VASB1/TS*) [47]. The bacteria we screened were different. For PBS, 11.8% of particles were degraded in 30 days, and *Pandoraea* and *Dyella* played a major role. The bacteria for PE degradation were *Acidovorax*, *Bdellovibrio*, and *Acinetobacter*.

Under the action of *Burkholderia-Caballeronia-Paraburkholderia*, the weight of PVC and PP decreased by 8.90% and 13.1%, respectively, within 30 days. Existing studies on the degradation of PVC have found that under the action of *Chaetomium globosum*, PVC can be degraded by ~9% after 28 days [48]. The main MP-degrading bacteria for PP is *Stenotrophomonas panacihumi PA3-2*, which can degrade PP by 18.4% in 40 days [49]. Our results show that the main degrading bacteria for PA, PLA, and PHA are *Novosphingobium*, *Dyella*, and *Burkholderia-Caballeronia-Paraburkholderia*, which degrade approximately 12.0%. The *Bacillus* sp. mentioned above can achieve 7% and 40% degradation rates for PA and PLA, respectively.

### 3.4. Degradation Mechanism

**Figure 6:**
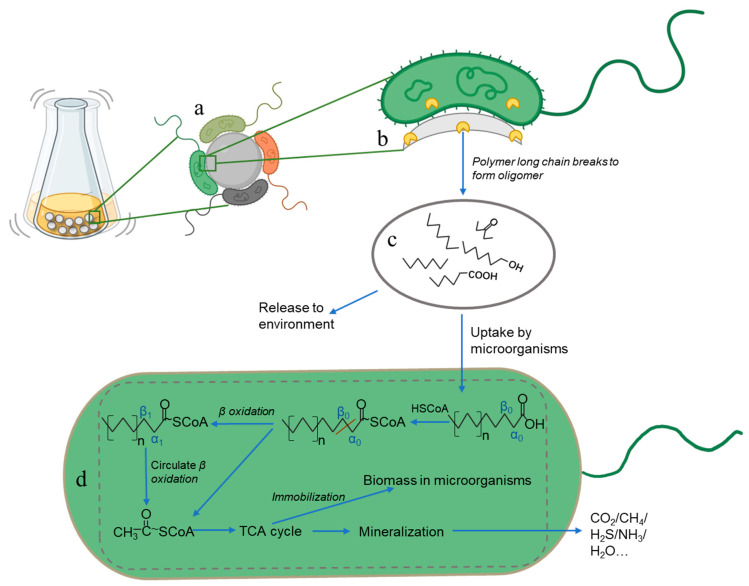
This represents the mechanism by which the microbial culture medium degrades microplastics. Subfigures (**a**) represents the microorganisms attach on the MPs surface to form biofilm. Subfigures (**b**) represents the microorganisms with plastic degrading ability secrete enzymes and other substances to attack the MP surface. Subfigures (**c**) represents some long chain polymers break to form oligomers and oxidize under the influence of oxygen to form carbonyl. Subfigures (**d**) represents some oligomers are ingested and assimilated by in vivo metabolism mechanism by microorganisms.

In the culture medium, first, some microbial aggregates composed of cells and extracellular polymers can adhere to each other or adhere to the surface of MPs to form complex biofilms [50,51,52] (Figure 6a). The biofilm includes one or more bacteria, which is a biological community with diversity in phylogeny and function [53]. Microbes in biofilms usually have the potential to metabolize a variety of substances, so the formation of biofilms plays a very important role in the microbial degradation of plastics. For example, using *Bacillus* sp. *JY36* to degrade PHB, PBS, PCL, and PBAT, the growth area of *Bacillus* sp. *JY36* was obvious after 7 days [54]; bacteria such as *Alcanivorax* sp., *Marinobacter* sp., and *Arenibacter* sp. were detected in biofilms formed on the surface of marine plastic fragments, and a large number of oxygen-containing functional groups were formed on the plastic surface, proving the potential of biofilms to degrade plastics [55].

Taking PE as an example, microorganisms with the ability to degrade MPs in the biofilms secrete extracellular and intracellular enzymes (Figure 6b), which break the end of the hydrolyzable chemical bond or molecular chain by attacking the plastic molecular chain, producing monomers, dimers, and other oligomers, leading to a reduction in the molecular weight of the MPs themselves [56,57]; meanwhile, PE is further oxidized under the action of oxygen to form hydroxyl-, carbonyl-, carboxyl- and other oxygen-containing functional groups (Figure 6c) [58]. The broken molecular chains and oligomers formed will be released into the surrounding environment, and some water-soluble short-chain intermediates in these depolymerized products will be recognized by the receptor and transported to the microbial body across the membrane as a carbon source for microbial metabolism and growth [59,60]. It is worth noting that some bacteria not only have the ability to degrade the polymer itself, but also have a strong ability to degrade the monomers, dimers, and other oligomers that make up the polymer [61].

The oligomer entering the microorganism will gradually remove the carbon atom on the plastic polymer molecular chain and degrade under the effect of the metabolic mechanism (mainly *β*-oxidation mechanism) in vivo [62] (Figure 6d). The *β*-oxidation mechanism starts from the combination of fatty acids produced after oligomer oxidation with coenzyme A (HSCoA) to produce fatty acetyl coenzyme A. Then, it goes through dehydrogenation, water addition, dehydrogenation, and sulfur hydrolysis. In the fatty acid, the connection of carbon atoms *α*0 and *β*0 is broken to produce acetyl coenzyme A and fatty acetyl coenzyme A with two carbon atoms removed. The cyclic reaction between coenzyme A and fatty acetyl coenzyme A with two carbon atoms removed completely oxidizes the fatty acid.

Acetyl coenzyme A enters the tricarboxylic acid cycle (TCA cycle) (Figure 6d). The TCA cycle is one of the important ways for microorganisms to metabolize organic substances and obtain energy for growth and development [63]. In the TCA cycle, acetyl coenzyme A generated from oligomers through a *β*-oxidation mechanism acts as a key intermediate to oxidize organic substances and produce adenine triphosphate (ATP) to provide energy for cells.

Finally, after a series of metabolism processes in vivo, the oligomers undergo mineralization (Figure 6d). The type of mineralization products of mineralization depends on the availability of oxygen: after mineralization in an aerobic environment, organisms mainly release CO_2_ and H_2_O, while under anoxic or anaerobic conditions, they produce CH_4_, NH_3_ or H_2_S [64].

### 3.5. Limitations

In this study, mangrove sediment was used to domesticate and degrade different MPs. By measuring the weight loss of different MPs before and after microbial degradation and comparing the weights with that of the control, the degradation effect of microorganisms on MPs can be judged intuitively by percentage, which is conducive to comparing the degradation ability of different strains horizontally. The weight loss method is currently a widely used method. However, there are some limitations; for example, the degradation phenomenon cannot be displayed directly and the decomposition rate is generally slow.

Appropriate improvements can also be made in the methods for screening MP-degrading bacteria, such as through directional acclimation of the in situ environment, stable isotope probe technology combined with laboratory culture, and construction of highly efficient MP-degrading bacteria based on synthetic microbiome, which can improve the efficiency of isolation of MP-degrading bacteria from the environment. In addition, the characteristics, optimization of degradation conditions, and degradation mechanism of different MP-degrading bacteria can also be studied in a follow-up of this study to further improve the degradation efficiency of MP-degrading bacteria.

## 4. Conclusions

According to our results, the biomass of the microbial culture medium after acclimation ranged from 88 to 699 mg/L. The bacterial growth on different MPs ranged from 0.03 to 0.09 OD 600 for the first generation to 0.009 to 0.081 OD 600 for the third generation. The weight loss method detected that the mass losses of PHB, PE, and PHA with large weight losses were 13.4%, 13.0%, and 12.7% after 30 days of degradation, respectively. The mass losses of PVC and PS were relatively low, at 8.90% and 9.10%, respectively. The degradation cycle t_1/2_ for the 11 kinds of MPs ranged from 67 to 116 days. As domestication progressed, *Pseudomonas* sp., *Pandoraea* sp., and *Dyella* sp. were able to grow well in the mixed strains. The possible mechanism of degradation is that microbial aggregates can adhere to the surface of MPs and form complex biofilms, secrete extracellular and intracellular enzymes, break the hydrolyzable chemical bonds or ends of molecular chains by attacking the molecular chains of the MPs, and produce monomers, dimers, and other oligomers, leading to a reduction in the molecular weight of the MPs. In the future, screening high degradation efficiency microbial communities and verifying degradation mechanisms should be given more attention.

## Figures and Tables

**Table 1:** Eleven MP degradation kinetic equations and coefficients.

Types	OD	R^2^	K	Degradation Kinetics	t_1/2_ (Day)
PVC	0.13	0.89	0.0074	W = W_0_ × 10^−0.007t^	93.34
PS	0.21	0.89	0.0061	W = W_0_ × 10^−0.006t^	114.1
PVA	0.47	0.89	0.0102	W = W_0_ × 10^−0.01t^	68.17
PA	0.68	0.80	0.0060	W = W_0_ × 10^−0.006t^	115.6
PHB	0.35	0.73	0.0102	W = W_0_ × 10^−0.01t^	67.98
PCL	0.24	0.90	0.0061	W = W_0_ × 10^−0.006t^	113.3
PBS	0.40	0.66	0.0069	W = W_0_ × 10^−0.007t^	100.6
PLA	0.26	0.88	0.0081	W = W_0_ × 10^−0.008t^	85.76
PP	0.82	0.76	0.0082	W = W_0_ × 10^−0.008t^	84.84
PE	0.12	0.80	0.0085	W = W_0_ × 10^−0.008t^	81.83
PHA	0.81	0.71	0.0067	W = W_0_ × 10^−0.007t^	103.6

## Data Availability

Data is contained within the article or supplementary material. The data presented in this study are available in *Biodeterioration of Microplastics by Bacteria Isolated from Mangrove Sediment* and supplementary material.

## Supplementary Materials

The following supporting information can be downloaded at: https://www.mdpi.com/article/10.3390/toxics11050432/s1, Table S1: Comparison of degradation and screening microorganisms of different microplastics. References [65,66,67,68,69,70,71,72,73,74,75,76,77,78,79,80,81] are cited in the supplementary materials.

## References

[1] Chae Y., An Y.-J. (2018). Current research trends on plastic pollution and ecological impacts on the soil ecosystem: A review. Environ. Pollut..

[2] Plastics E. (2022). An Analysis of European Plastics Production, Demand and Waste Data. Plastics—The Facts.

[3] OECD (2022). Global Plastics Outlook.

[4] Ren S.-Y., Ni H.-G. (2022). A method for measuring the emissions of in situ agricultural plastic film microplastics by ultraviolet and mechanical abrasion. Sci. Total Environ..

[5] Ren S.-Y., Sun Q., Ni H.-G., Wang J. (2020). A minimalist approach to quantify emission factor of microplastic by mechanical abrasion. Chemosphere.

[6] Andrady A.L. (2011). Microplastics in the marine environment. Mar. Pollut. Bull..

[7] Alimba C.G., Faggio C. (2019). Microplastics in the marine environment: Current trends in environmental pollution and mechanisms of toxicological profile. Environ. Toxicol. Pharmacol..

[8] Allen S., Allen D., Phoenix V.R., Le Roux G., Jimenez P.D., Simonneau A., Binet S., Galop D. (2019). Atmospheric transport and deposition of microplastics in a remote mountain catchment. Nat. Geosci..

[9] Liu Q.-M., Liang H.-T., Xi G.-L., Hu X., Ge J. (2019). Microplastic Pollution of the Beaches in Xiamen Bay, China. Huan Jing Ke Xue = Huanjing Kexue.

[10] Ren S.-Y., Kong S.-F., Ni H.-G. (2021). Contribution of mulch film to microplastics in agricultural soil and surface water in China. Environ. Pollut..

[11] Bour A., Haarr A., Keiter S., Hylland K. (2018). Environmentally relevant microplastic exposure affects sediment-dwelling bivalves. Environ. Pollut..

[12] Kaposi K.L., Mos B., Kelaher B.P., Dworjanyn S.A. (2014). Ingestion of Microplastic Has Limited Impact on a Marine Larva. Environ. Sci. Technol..

[13] Zhang C., Chen X.H., Wang J.T., Tan L.J. (2017). Toxic effects of microplastic on marine microalgae *Skeletonema costatum*: Interactions between microplastic and algae. Environ. Pollut..

[14] de Ruijter V.N., Redondo-Hasselerharm P.E., Gouin T., Koelmans A.A. (2020). Quality Criteria for Microplastic Effect Studies in the Context of Risk Assessment: A Critical Review. Environ. Sci. Technol..

[15] Wang W., Ndungu A.W., Li Z., Wang J. (2017). Microplastics pollution in inland freshwaters of China: A case study in urban surface waters of Wuhan, China. Sci. Total Environ..

[16] Rillig M.C., Ingraffia R., Machado A.A.d.S. (2017). Microplastic Incorporation into Soil in Agroecosystems. Front. Plant Sci..

[17] Gewert B., Plassmann M.M., MacLeod M. (2015). Pathways for degradation of plastic polymers floating in the marine environment. Environ. Sci. Process. Impacts.

[18] Auta H.S., Emenike C.U., Fauziah S.H. (2017). Screening of Bacillus strains isolated from mangrove ecosystems in Peninsular Malaysia for microplastic degradation. Environ. Pollut..

[19] Amobonye A., Bhagwat P., Singh S., Pillai S. (2021). Plastic biodegradation: Frontline microbes and their enzymes. Sci. Total Environ..

[20] Tokiwa Y., Calabia B.P., Ugwu C.U., Aiba S. (2009). Biodegradability of Plastics. Int. J. Mol. Sci..

[21] Ramis X., Cadenato A., Salla J.M., Morancho J.M., Valles A., Contat L., Ribes A. (2004). Thermal degradation of polypropylene/starch-based materials with enhanced biodegradability. Polym. Degrad. Stab..

[22] Flemming H.C. (1998). Relevance of biofilms for the biodeterioration of surfaces of polymeric materials. Polym. Degrad. Stab..

[23] Peng B.Y., Su Y.M., Chen Z.B., Chen J.B., Zhou X.F., Benbow M.E., Griddle C.S., Wu W.M., Zhang Y.L. (2019). Biodegradation of Polystyrene by Dark (*Tenebrio obscurus*) and Yellow (Tenebrio molitor) Mealworms (Coleoptera: Tenebrionidae). Environ. Sci. Technol..

[24] Atwood T.B., Connolly R.M., Almahasheer H., Carnell P.E., Duarte C.M., Lewis C.J.E., Irigoien X., Kelleway J.J., Lavery P.S., Macreadie P.I. (2017). Global patterns in mangrove soil carbon stocks and losses. Nat. Clim. Chang..

[25] Hamilton S.E., Casey D. (2016). Creation of a high spatio-temporal resolution global database of continuous mangrove forest cover for the 21st century (CGMFC-21). Glob. Ecol. Biogeogr..

[26] Mohan A.J., Sekhar V.C., Bhaskar T., Nampoothiri K.M. (2016). Microbial assisted High Impact Polystyrene (HIPS) degradation. Bioresour. Technol..

[27] Auta H.S., Emenike C.U., Jayanthi B., Fauziah S.H. (2018). Growth kinetics and biodeterioration of polypropylene microplastics by *Bacillus* sp and *Rhodococcus* sp isolated from mangrove sediment. Mar. Pollut. Bull..

[28] Sun Q., Ren S.-Y., Ni H.-G. (2022). Effects of microplastic sorption on microbial degradation of halogenated polycyclic aromatic hydrocarbons in water. Environ. Pollut..

[29] Alaribe F.O., Agamuthu P. (2015). Assessment of phytoremediation potentials of Lantana camara in Pb impacted soil with organic waste additives. Ecol. Eng..

[30] Rujnic-Sokele M., Pilipovic A. (2017). Challenges and opportunities of biodegradable plastics: A mini review. Waste Manag. Res..

[31] Vasquez-Murrieta M.S., Hernandez-Hernandez O.J., Cruz-Maya J.A., Cancino-Diaz J.C., Jan-Roblero J. (2016). Approaches for Removal of PAHs in Soils: Bioaugmentation, Biostimulation and Bioattenuation. Soil Contamination-Current Consequences and Further Solutions.

[32] Sivan A., Szanto M., Pavlov V. (2006). Biofilm development of the polyethylene-degrading bacterium *Rhodococcus ruber*. Appl. Microbiol. Biotechnol..

[33] Skariyachan S., Patil A.A., Shankar A., Manjunath M., Bachappanavar N., Kiran S. (2018). Enhanced polymer degradation of polyethylene and polypropylene by novel thermophilic consortia of *Brevibacillus* sps. and *Aneurinibacillus* sp. screened from waste management landfills and sewage treatment plants. Polym. Degrad. Stab..

[34] Pettigrew C.A., Breen A., Corcoran C., Sayler G.S. (1990). Chlorinated Biphenyl Mineralization by Individual Populations and Consortia of Fresh-Water Bacteria. Appl. Environ. Microbiol..

[35] Jeon H.J., Kim M.N. (2013). Biodegradation of poly(L-lactide) (PLA) exposed to UV irradiation by a mesophilic bacterium. Int. Biodeterior. Biodegrad..

[36] Cohen J. (1988). Statistical Power Analysis for the Behavioral Sciences.

[37] Wei R., Zimmermann W. (2017). Microbial enzymes for the recycling of recalcitrant petroleum-based plastics: How far are we?. Microb. Biotechnol..

[38] Lambert S., Wagner M. (2017). Environmental performance of bio-based and biodegradable plastics: The road ahead. Chem. Soc. Rev..

[39] Haney C.H., Samuel B.S., Bush J., Ausubel F.M. (2015). Associations with rhizosphere bacteria can confer an adaptive advantage to plants. Nat. Plants.

[40] Ee R., Yong D., Lim Y.L., Yin W.F., Chan K.G. (2015). Complete genome sequence of oxalate-degrading bacterium *Pandoraea vervacti* DSM 23571(T). J. Biotechnol..

[41] Wang S.Z., Yin Y.A., Wang J.L. (2018). Microbial degradation of triclosan by a novel strain of *Dyella* sp. Appl. Microbiol. Biotechnol..

[42] Abdel-Motaal F.F., El-Sayed M.A., El-Zayat S.A., Ito S. (2014). Biodegradation of poly (epsilon-caprolactone) (PCL) film and foam plastic by *Pseudozyma japonica* sp nov.; a novel cutinolytic ustilaginomycetous yeast species. 3 Biotech.

[43] Wang Z.Y., Lin X.D., An J., Ren C., Yan X. (2013). Biodegradation of Polyhydroxybutyrate Film by Pseudomonas mendocina DS04-T. Polym. -Plast. Technol. Eng..

[44] Hoang K.C., Tseng M., Shu W.J. (2007). Degradation of polyethylene succinate (PES) by a new thermophilic Microbispora strain. Biodegradation.

[45] Xu L., Fan J.-H., Ma L.-M. (2009). Progress on Degradation of Microorganism by PVA. Environ. Sci. Technol..

[46] Hayase N., Yano H., Kudoh E., Tsutsumi C., Ushio K., Miyahara Y., Tanaka S., Nakagawa K. (2004). Isolation and characterization of poly(butylene succinate-co-butylene adipate)-degrading microorganism. J. Biosci. Bioeng..

[47] Shahnawaz M., Sangale M.K., Ade A.B. (2016). Rhizosphere of Avicennia marina (Forsk.) Vierh. as a landmark for polythene degrading bacteria. Environ. Sci. Pollut. Res..

[48] Vivi V.K., Martins-Franchetti S.M., Attili-Angelis D. (2019). Biodegradation of PCL and PVC: Chaetomium globosum (ATCC 16021) activity. Folia Microbiol..

[49] Jeon H.J., Kim M.N. (2016). Isolation of mesophilic bacterium for biodegradation of polypropylene. Int. Biodeterior. Biodegrad..

[50] Flemming H.C., Wingender J., Szewzyk U., Steinberg P., Rice S.A., Kjelleberg S. (2016). Biofilms: An emergent form of bacterial life. Nat. Rev. Microbiol..

[51] Cho J.Y., Park S.L., Kim S.H., Jung H.J., Cho D., Kim B.C., Bhatia S.K., Gurav R., Park S.H., Park K. (2022). Novel Poly(butylene adipate-co-terephthalate)-degrading *Bacillus* sp. JY35 from wastewater sludge and its broad degradation of various bioplastics. Waste Manag..

[52] Cho J.Y., Park S.L., Lee H.J., Kim S.H., Suh M.J., Ham S., Bhatia S.K., Gurav R., Park S.H., Park K. (2021). Polyhydroxyalkanoates (PHAs) degradation by the newly isolated marine *Bacillus* sp. JY14. Chemosphere.

[53] Rummel C.D., Jahnke A., Gorokhova E., Kuhnel D., Schmitt-Jansen M. (2017). Impacts of Biofilm Formation on the Fate and Potential Effects of Microplastic in the Aquatic Environment. Environ. Sci. Technol. Lett..

[54] Cho J.Y., Kim S.H., Cho D.H., Jung H.J., Kim B.C., Bhatia S.K., Gurav R., Lee J., Park S.H., Park K. (2022). Simultaneous monitoring of each component on degradation of blended bioplastic using gas chromatography-mass spectrometry. Anal. Biochem..

[55] Delacuvellerie A., Cyriaque V., Gobert S., Benali S., Wattiez R. (2019). The plastisphere in marine ecosystem hosts potential specific microbial degraders including *Alcanivorax borkumensis* as a key player for the low-density polyethylene degradation. J. Hazard. Mater..

[56] Park S.L., Cho J.Y., Choi T.R., Song H.S., Bhatia S.K., Gurav R., Park S.H., Park K., Joo J.C., Hwang S.Y. (2021). Improvement of polyhydroxybutyrate (PHB) plate-based screening method for PHB degrading bacteria using cell-grown amorphous PHB and recovered by sodium dodecyl sulfate (SDS). Int. J. Biol. Macromol..

[57] Park S.L., Cho J.Y., Kim S.H., Bhatia S.K., Gurav R., Park S.H., Park K., Yang Y.H. (2021). Isolation of *Microbulbifer* sp. SOL66 with High Polyhydroxyalkanoate-Degrading Activity from the Marine Environment. Polymers.

[58] Gong J.X., Kong T.T., Li Y.Q., Li Q.J., Li Z., Zhang J.F. (2018). Biodegradation of Microplastic Derived from Poly(ethylene terephthalate) with Bacterial Whole-Cell Biocatalysts. Polymers.

[59] Shah A.A., Hasan F., Hameed A., Ahmed S. (2008). Biological degradation of plastics: A comprehensive review. Biotechnol. Adv..

[60] Gao R., Liu R., Sun C. (2022). A marine fungus Alternaria alternata FB1 efficiently degrades polyethylene. J. Hazard. Mater..

[61] Liu J., Xu G., Dong W., Xu N., Xin F., Ma J., Fang Y., Zhou J., Jiang M. (2018). Biodegradation of diethyl terephthalate and polyethylene terephthalate by a novel identified degrader *Delftia* sp WL-3 and its proposed metabolic pathway. Lett. Appl. Microbiol..

[62] Lucas N., Bienaime C., Belloy C., Queneudec M., Silvestre F., Nava-Saucedo J.E. (2008). Polymer biodegradation: Mechanisms and estimation techniques. Chemosphere.

[63] Pathak V.M. (2017). Navneet. Review on the current status of polymer degradation: A microbial approach. Bioresour. Bioprocess..

[64] Mohee R., Unmar G.D., Mudhoo A., Khadoo P. (2008). Biodegradability of biodegradable/degradable plastic materials under aerobic and anaerobic conditions. Waste Manag..

[65] Kumari A., Chaudhary D.R., Jha B. (2019). Destabilization of polyethylene and polyvinylchloride structure by marine bacterial strain. Environ. Sci. Pollut. Res..

[66] Mor R., Sivan A. (2008). Biofilm formation and partial biodegradation of polystyrene by the actinomycete Rhodococcus ruber. Biodegradation.

[67] Muenmee S., Chiemchaisri W., Chiemchaisri C. (2015). Microbial consortium involving biological methane oxidation in relation to the biodegradation of waste plastics in a solid waste disposal open dump site. Inter. Biodeterior. Biodegrad..

[68] Kang C.Y., Kim S.S., Kim S.J., Lee J. (2017). The Significant Influence of Bacterial Reaction on Physico-Chemical Property Changes of Biodegradable Natural and Synthetic Polymers Using Escherichia coli. Polymers.

[69] Sudhakar M., Priyadarshini C., Doble M., Murthy P.S., Venkatesan R. (2007). Marine bacteria mediated degradation of nylon 66 and 6. Inter. Biodeterior. Biodegrad..

[70] Tansengco M., Dogma I. (1999). Microbial degradation of poly-beta-hydroxybutyrate using landfill soils. Acta Biotechnol..

[71] Abou-Zeid D.M., Muller R.J., Deckwer W.D. (2001). Degradation of natural and synthetic polyesters under anaerobic conditions. J. Biotechnol..

[72] Oda Y., Asari H., Urakami T., Tonomura K. (1995). Microbial-degradation of poly(3-hydroxybutyrate) and polycaprolactone by filamentous Fungi. J. Ferment. Bioeng..

[73] Xu S., Yamaguchi T., Osawa S., Suye S.-I. (2007). Biodegradation of poly (epsilon-caprolactone) film in the presence of *Lysinibacillus* sp. 70038 and characterization of the degraded film. Biocontrol Sci..

[74] Abe M., Kobayashi K., Honma N., Nakasaki K. (2010). Microbial degradation of poly(butylene succinate) by Fusarium solani in soil environments. Polym. Degrad. Stab..

[75] Jia H., Zhang M., Weng Y., Li C. (2020). Isolation and characterization of polylactic acid-degrading bacteria and their enzyme production and degradation characteristics. Fine Chem..

[76] Nair N.R., Sekhar V.C., Nampoothiri K.M. (2016). Augmentation of a Microbial Consortium for Enhanced Polylactide (PLA) Degradation. Indian J. Microbiol..

[77] Tomita K., Nakajima T., Kikuchi Y., Miwa N. (2004). Degradation of poly(L-lactic acid) by a newly isolated thermophile. Polym. Degrad. Stab..

[78] Kim M.N., Kim W.G., Weon H.Y., Lee S.H. (2008). Poly(L-lactide)-degrading activity of a newly isolated bacterium. J. App. Polym. Sci..

[79] Balasubramanian V., Natarajan K., Hemambika B., Ramesh N., Sumathi C.S., Kottaimuthu R., Kannan V.R. (2010). High-density polyethylene (HDPE)-degrading potential bacteria from marine ecosystem of Gulf of Mannar, India. Lett. App. Microbiol..

[80] Park S.Y., Kim C.G. (2019). Biodegradation of micro-polyethylene particles by bacterial colonization of a mixed microbial consortium isolated from a landfill site. Chemosphere.

[81] Kowalczyk A., Chyc M., Ryszka P., Latowski D. (2016). Achromobacter xylosoxidans as a new microorganism strain colonizing high-density polyethylene as a key step to its biodegradation. Environ. Sci. Pollut. Res..

