# Supplementary material for: Biodeterioration of Microplastics by Bacteria Isolated from Mangrove Sediment
Source: Toxics. 2023 May 5;11(5):432. doi: 10.3390/toxics11050432 (PMC10222261; doi:10.3390/toxics11050432)
Supplement: Supplementary file 1 [file toxics-11-00432-s001.zip › toxics-2321152-supplementary.pdf]

Supplementary Materials for:

Biodeterioration of microplastics by bacteria isolated from mangrove sediment

Shu-Yan Ren and Hong-Gang Ni*

School of Urban Planning and Design, Peking University Shenzhen Graduate School, Shenzhen 518055, China.

Corresponding Author:

Phone: +86-755-26033017. Fax: +86-755-26033254. E-mail: nihg@pkusz.edu.cn

Table S1 Degradation and screening microorganisms of different microplastics.

This study						Other studies			References
Types	OD	Biomass (mg/L)	Duration of degradation (d)	Gravimetric weight loss (%)	Major degrading bacteria	Duration of degradation (d)	Gravimetric weight loss (%)	Major degrading bacteria	
PVC	0.13	587.9	30	8.9	*Burkholderia-Caballeronia-Paraburkholderia, Pandoraea*	28	9.00	*Chaetomium globosum*	[48]
						90	0.26	*Bacillus sp. AIIW2*	[65]

PS	0.21	386.1	30	9.2	*Hyphomicrobium, f_Xanthobacteraceae_Unclassified, Pandoraea, f_Xanthobacteraceae_Unclassified*	30	12.4	/	[26]
						30	40.2	*Pseudozyma japonica Y7-09*	[42]
						56	0.80	*Rhodococcus ruber C208*	[66]
PP	0.82	87.63	30	13	*Burkholderia-Caballeronia-Paraburkholderia,*	40	18.4	*Stenotrophomonas panacihumi PA3-2*	[49]

					Pandoraea	40	6.40	*Rhodococcus sp. 36*	[27]
					f_Xanthobacteraceae_Unclassified	357	6.70	*Methylobacter sp. and Methylocella sp.*	[67]
PVA	0.47	158.7	30	12	*Pseudomonas, Burkholderia-Caballeronia-Paraburkholderia, Pandoraea*	12	100	*Penicilllium sp. WAH02-21*	[45]
						46	75.0	*cardiobacterium sp.SB68*	[45]
PA	0.68	133.6	30	12	*Novosphingobium, f_Rhizobiaceae_U*	30	/	*Escherichia coli*	[68]

					nclassified, Achromobacter				
						90	7.00	*Bacillus cereus, Bacillus sphericus,*	[69]
PHB	0.35	241.8	30	13	*f_Xanthobacteraceae_Unclassified, Dyella, Pandoraea*	52h	100	*Pseudomonas mendocina DS04-T*	[43]
						7	92.0	*Aspergillus 39*	[70]
						63	32.0	*Vibrio furnisii and Brevundimonas vesicularis*	[71]

PCL	0.24	372.2	30	9.7	*Pandoraea, f_Xanthobacteraceae_Unclassified*	7	100	*Micorbispora rosea subsp. Taiwanensis HS 45-1*	[44]
						10	10.0	*Paecilomyces lilacinus D218*	[72]
						30	9.00	*Lysinibacillus sp. 70038*	[73]
PBS	0.40	190.5	30	12	*Pandoraea, Dyella*	14	90.2	*Bacillus pumilus 1-A*	[46]
						14	2.80	*Fusarium solani WF-6*	[74]
PLA	0.26	299.5	30	12	*Dyella, Allorhizobium-Neorhizobium-*				

Pararhizobium-Rhizobium, Pandoraea				
	5	29.4	*Pseudomonas mendocina*	[75]
	15	26.9	*Cladosporium sphaerospermum, Rhodotorula mucilaginosa, Penicillium chrysogenum and Serratia marcescens*	[76]

						20	25.0	*Geobacillus thermocatenulatus*	[77]
						40	40.0	*Bacillus licheniformis*	[78]
PE	0.12	699.8	30	13	*Acidovorax, Bdellovibrio, Acinetobacter*	28	5.80-10.9	*Enterobacter asburiae, Bacillus*	[27]
						30	15.2	*Pseudomonas sp. GMB7*	[79]
						60	87.5	*Bacillus cereus VASB1/TS*	[47]
						60	14.7	*Bacillus sp. And Paenibacillus sp.*	[80]

						150	6.10	*Achromobacter xylosoxidans*	[81]
PHA	0.81	89.63	30	13	*Burkholderia-Caballeronia-Paraburkholderia, Klebsiella*				
